# Acquired microstomia after burns and trauma: a pathoanatomic classification based on the dominant restrictive layer

**DOI:** 10.1093/burnst/tkag062

**Published:** 2026-09-10

**Authors:** Huimin Fang, Xingran Liu, Kai Liu, Qingfeng Li, Ru-Lin Huang

**Affiliations:** Department of Plastic and Reconstructive Surgery, Shanghai Ninth People’s Hospital, Shanghai Jiao Tong University School of Medicine, 639 Zhizaoju Road, Shanghai 200011, China; Shanghai Institute for Plastic and Reconstructive Surgery, 1908 Gaoke Road, Shanghai 200125, China; Department of Plastic and Reconstructive Surgery, Shanghai Ninth People’s Hospital, Shanghai Jiao Tong University School of Medicine, 639 Zhizaoju Road, Shanghai 200011, China; Shanghai Institute for Plastic and Reconstructive Surgery, 1908 Gaoke Road, Shanghai 200125, China; Department of Plastic and Reconstructive Surgery, Shanghai Ninth People’s Hospital, Shanghai Jiao Tong University School of Medicine, 639 Zhizaoju Road, Shanghai 200011, China; Shanghai Institute for Plastic and Reconstructive Surgery, 1908 Gaoke Road, Shanghai 200125, China; Department of Plastic and Reconstructive Surgery, Shanghai Ninth People’s Hospital, Shanghai Jiao Tong University School of Medicine, 639 Zhizaoju Road, Shanghai 200011, China; Shanghai Institute for Plastic and Reconstructive Surgery, 1908 Gaoke Road, Shanghai 200125, China; Department of Plastic and Reconstructive Surgery, Shanghai Ninth People’s Hospital, Shanghai Jiao Tong University School of Medicine, 639 Zhizaoju Road, Shanghai 200011, China; Shanghai Institute for Plastic and Reconstructive Surgery, 1908 Gaoke Road, Shanghai 200125, China

**Letter to the Editor**,

Acquired microstomia presents a shared phenotype characterized by a reduced oral aperture and restricted mouth opening; however, this phenotype may result from different types of tissue injury. Conventional post-burn microstomia is usually caused by cutaneous or perioral soft-tissue loss and scar contracture, most commonly following thermal, flame, or electrical burns. Conversely, patients with caustic injury, tumour resection, or chronic mucosal injury may develop microstomia primarily driven by intraoral mucosal deficiency, vestibular obliteration, and mucosal scar contracture. These two patterns differ in anatomic location as well as tissue characteristics, healing behaviour, and reconstructive requirements. Therefore, the same small-mouth phenotype should not automatically lead to the same surgical strategy.

In surgical practice, definitive diagnosis and treatment planning are generally based on pathology and disease staging because similar clinical presentations may arise from fundamentally different disease processes. The same principle could apply to plastic and reconstructive surgery. In managing acquired microstomia, we have observed that a similar small-mouth phenotype may arise from injury to different tissue types, yielding fundamentally different pathologic and anatomic bases that require distinct reconstructive strategies. We propose an anatomic–pathologic framework in which acquired microstomia is classified into three types: Type I, with predominant perioral cutaneous soft-tissue deficiency; Type II, with predominant intraoral mucosal lining deficiency; Type III, with predominant perioral cutaneous soft-tissue and intraoral mucosal lining deficiency. This proposal is not simply a new label for microstomia, but a diagnostic framework that aligns assessment with the underlying pathology. Evaluation of a microstomia patient should not end with recognition of the phenotype alone. Instead, three sequential questions should be addressed: (i) What caused the deformity? (ii) How severe is the functional and aesthetic impairment? and (iii) Which tissue layer requires reconstruction? A stepwise approach based on aetiologic, functional–aesthetic, and anatomic–pathologic assessment may help ensure that the treatment targets the true reconstructive defect, including defects arising from prior surgery, rather than appearance alone.

This concept was shaped partly by our experience with caustic injury-related microstomia. We encountered patients with microstomia caused by intraoral lining deficiency who had previously undergone commissuroplasty at other institutions, with subsequent worsening of mouth opening limitation. We observed that in some patients with severe impairment, restricted mouth opening was driven by deeper intraoral scar contracture, vestibular shallowing or obliteration, and lining deficiency [1]. In such patients, lateral enlargement of the commissure may fail to address the true intraoral mucosal lining deficiency and may increase tension on an already deficient mucosal lining. Building on this observation, we propose an anatomic–pathologic classification framework applicable to a broader spectrum of acquired microstomia.

Within this framework, acquired microstomia can be divided into three types (Figure 1). Type I, characterized by perioral soft-tissue deficiency, is most commonly seen after thermal, electrical, or flame burns with scar contracture. Typical findings include reduced oral width, commissural malposition, and loss of perioral soft-tissue pliability, while the intraoral lining remains relatively preserved. In this group, restricted mouth opening is primarily caused by tethering of the external perioral soft-tissue envelope [2]. Conversely, Type II, characterized by intraoral lining deficiency, is most commonly associated with caustic ingestion, chronic mucosal damage from long-term areca nut chewing, or loss of intraoral lining after tumour resection. Although these patients may also present with a reduced oral aperture and limited mouth opening, the principal restrictive element is scar contracture of the labial lining, buccal lining, gingivobuccal sulcus, and oral vestibule. On examination, the vestibule is often shallow or obliterated, the lining is stiff, and the vermilion may be turned inward. Type III, characterized by combined perioral soft-tissue and intraoral lining deficiency, is seen in patients with severe burns, complex trauma, or extensive caustic injury involving both the perioral skin and intraoral mucosa. In this group, limited mouth opening results from the combined effects of external perioral scar contracture and intraoral lining contracture. On examination, patients may present with reduced oral width, commissural distortion, poor perioral soft-tissue pliability, and varying degrees of vestibular shallowing or obliteration. Recognition of both components is essential for appropriate assessment and individualized treatment planning. This grouping is based on the dominant anatomical–pathological level of restriction rather than aetiology alone, and the specific reconstructive method should still be individualized according to the cause, extent of injury, and tissue characteristics.

**Figure 1:**
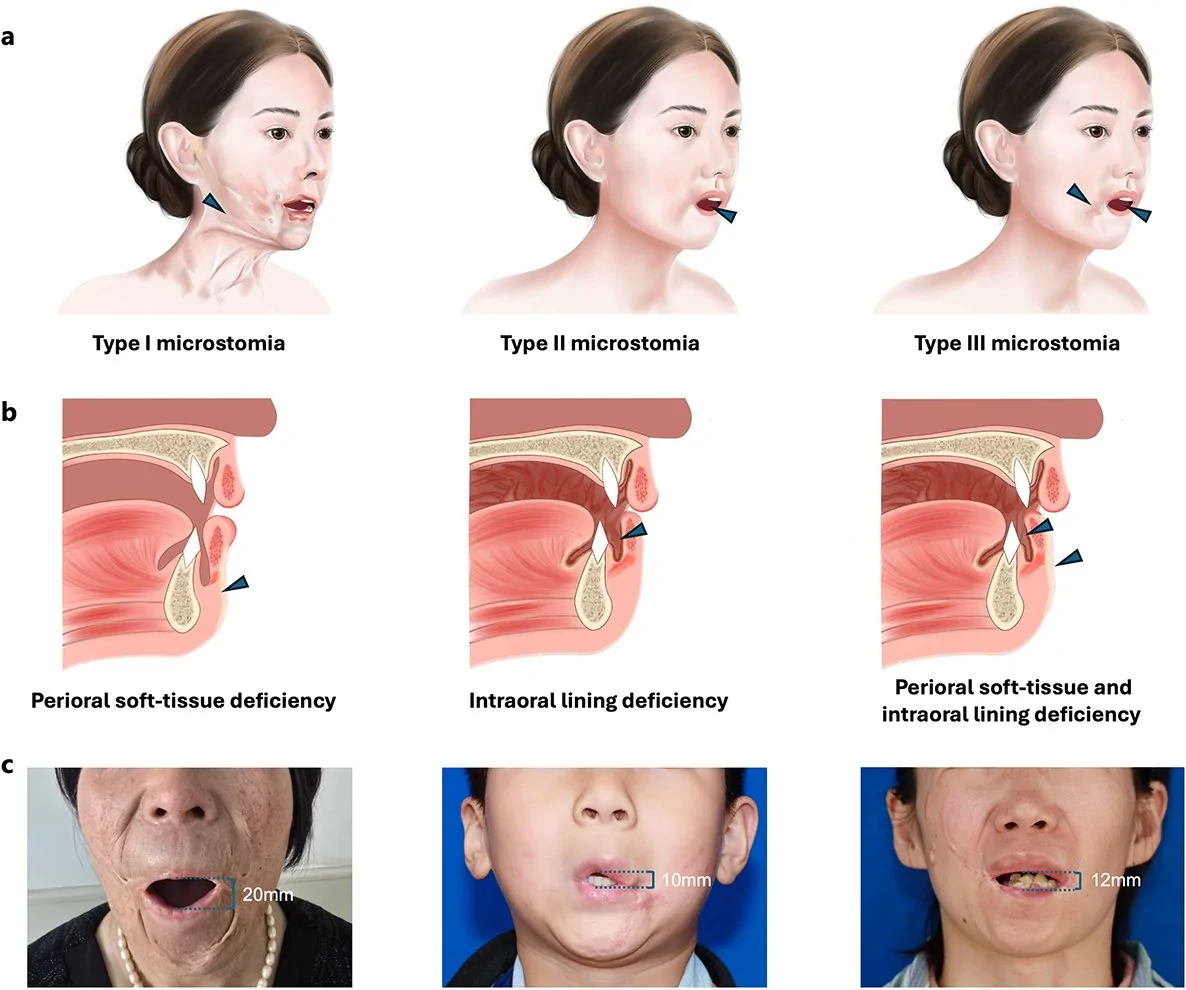
Proposed anatomical–pathological classification of microstomia. (**a**) Schematic external views of the three types of microstomia. Type I microstomia is characterized predominantly by perioral soft-tissue deficiency, Type II by intraoral lining deficiency, and Type III by combined perioral soft-tissue and intraoral lining deficiency. (**b**) Corresponding cross-sectional schematic illustrations showing the principal anatomical level of restriction in each type. Arrowheads indicate the dominant restrictive component. (**c**) Representative clinical photographs of patients with Type I (20 mm), Type II (10 mm), and Type III (12 mm) microstomia, respectively

This classification is intended to facilitate anatomical–pathological assessment rather than to prescribe a fixed reconstructive algorithm. It may help direct the surgeon’s attention to the principal layer requiring release and reconstruction. However, the specific reconstructive method should be individualized according to the aetiology, severity, extent of scarring, tissue quality, and prior treatment history. Depending on these factors, reconstruction may involve local flaps, mucosal flaps, skin grafting, or free-tissue transfer [3, 4]. Long-term postoperative stenting and mouth opening exercises may be important to reduce recurrent contracture, and severe cases may require multidisciplinary perioperative management [5]. The key point is that a similar small-mouth phenotype does not necessarily reflect the same underlying defect; therefore, treatment should be based first on the involved anatomical level and then tailored to the specific clinical context.

The proposed classification shifts the diagnostic focus from phenotype to anatomic–pathologic characterization. Its significance lies not merely in introducing new terminology but in emphasizing that diagnosis should identify both the cause of the deformity and the specific tissue layer requiring reconstruction. Such an approach may better align anatomical–pathological assessment with individualized reconstructive planning, thereby reducing the risk of inappropriate treatment and improving the precision of functional restoration. This framework should therefore be regarded as a practical diagnostic aid rather than a rigid algorithm. Future studies should evaluate its reproducibility among surgeons and determine whether classification-guided reconstruction improves mouth opening, oral competence, recurrence rates, and patient-reported outcomes. Nonetheless, we believe that this anatomy-based framework, together with a reconstructive sequence integrating aetiologic, functional–aesthetic, and anatomic–pathologic assessment, may provide a more rational basis for individualized evaluation and treatment planning in acquired microstomia.

## Data Availability

The data supporting the findings of this study are available from the corresponding author on reasonable request, subject to patient confidentiality and institutional regulations.
